# Supplementary material for: Cell Cycle Regulation of Hippocampal Progenitor Cells in Experimental Models of Depression and after Treatment with Fluoxetine
Source: Int J Mol Sci. 2021 Oct 30;22(21):11798. doi: 10.3390/ijms222111798 (PMC8584049; doi:10.3390/ijms222111798)
Supplement: Supplementary file 1 [file ijms-22-11798-s001.zip › ijms-1419815-supplementary.pdf]

SUPPLEMENTARY MATERIALS

	Mon	Tue	Wed	Thu	Fri	Sat	Sun
9h - 10h	Confinement	Inaccessible Food	Empty Bottle	Startle Noise	Confinement		
10h - 11h			Strobe Lights		Tilted Cage		
11h - 12h	Strobe Lights	Overcrowding					
12h - 13h							
13h - 14h			Tilted cage	Overcrowding	Strobe Lights		
14h - 15h	Tilted cage	Startle Noise					
15h - 18h			Confinement				
O/N	Food Deprivation	water Deprivation + O/N Illumination	Strobe lights (low frequency)	Wet Bed	Reverse Light/dark + Tilted Cage		

Supplementary Figure S1: *uCMS protocol* (one week example). The order of each stressor (confinement, strobe lights, tilted cage, food/water deprivation, overcrowding, O/N illumination, empty bottle, startle noise, wet bed, reverse light/dark hours) changed every week throughout the 6 weeks protocol. During the last 2 weeks of the protocol, Fluoxetine was administered in the afternoon, between 2 and 4 p.m.

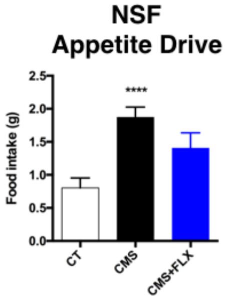

Supplementary Figure S2: *Food intake assessment after the Novelty Suppressed Feeding Test.* To validate significant changes in latency to feed assessed by this behavioral paradigm, alterations in appetite drive must be characterized. In this way, a significant increase in latency to feed (anxiety-like behavior) can only be validated when not accompanied by decreased appetite drive. Conversely, a significant increase in latency to feed (an anxiogenic effect) may only be considered when not associated with a significant decrease in appetite drive. Although uCMS-exposed animals presented significantly higher appetite drive when compared to CT (CT=0.8025 ± 0.1409 g vs uCMS=1.872 ± 0.1463 g, t_{18}= 5.264, $p<0.0001$), they also showed an increased latency to feed, thus not configuring any of the above-mentioned situations. Error bars denote SEM. *Denotes the effect of uCMS-exposure; #Denotes the effect of fluoxetine compared with untreated uCMS-exposed animals. ****$p<0.0001$. Abbreviations: uCMS: unpredictable chronic mild stress; FLX: fluoxetine.

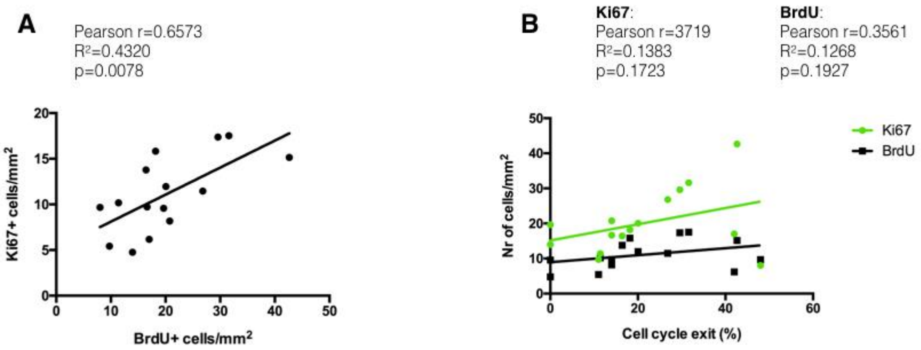

Supplementary Figure S3: A. Pearson correlation between the numbers of KI67 and BrdU positive cells per mm^2 in the DG. **B.** Pearson correlation between the numbers of BrdU positive or Ki67 positive cells per mm^2 and the % of cells that have exited the cell cycle.

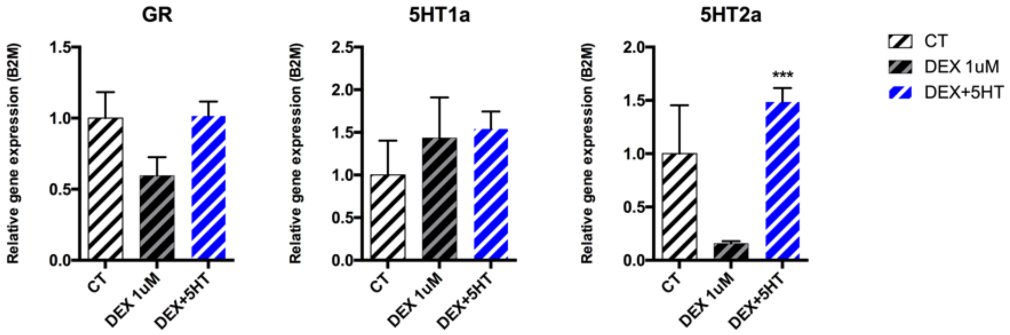

Supplementary Figure S4: Expression of glucocorticoid receptor and 5-HT receptors in the neurospheres, measured by qRT-PCR. DEX treatment did not induce statistically significant changes in the levels of GR, 5-HT1a or 5-HT2a. 5-HT could only significantly increase the expression of 5-HT2a when compared to DEX-treated cells (t_4=11.37; p=0.0003). Error bars denote SEM. *Denotes the effect of fluoxetine compared with untreated uCMS-exposed animals. ***p<0.001. Abbreviations: CT: Control; DEX: Dexamethasone, 5HT: serotonin, GR: glucocorticoid receptor; 5HT1a: serotonin receptor 1a; 5HT2a: serotonin receptor 2a.

Supplementary Table S1: list of primary and secondary antibodies used with catalogue numbers and RRIDs.

Designation	Catalogue nr	RRID
Anti-Bromodeoxyuridin (BrdU)	Agilent Cat# M0744	AB_10013660
Anti-Doublecortin (DCX)	Millipore Cat. No. AB2253	-
Anti-Ki67	Millipore Cat# AB9260	AB_2142366
Anti-Nestin	Millipore Cat# MAB353	AB_94911
Anti-GFAP	Agilent Cat# Z0334	AB_10013382
Anti-β-3 tubulin	Sigma-Aldrich Cat# T8660	AB_477590
Anti-O4	R and D Systems Cat# MAB1326	AB_357617
Anti-mouse Alexa-fluor® 488	Catalog # A-11029	-
Anti-rabbit Alexa-fluor® 594	Catalog # A-11037	-
Anti-mouse Alexa-fluor® 594	Catalog # A-11005	-
Anti-rabbit Alexa-fluor® 488	Catalog # A-11008	-
Anti-rat Alexa-fluor® 594	Catalog # A-11007	-
Anti-mouse Alexa-fluor® 647	Catalog # A-31571	-

Supplementary Table S2: Sense and antisense sequences of oligonucleotide primers used in the quantitative real time polymerase chain reaction (qRT-PCR) and their corresponding product size.

Gene symbol	Sense	Antisense	Product size (bp)
Cyclin D1	CGCGTACCCTGACACCAATCT	CTTCTGCTCCTCGCAGACCTCTA	172
Cyclin D2	ACACCGATGTGGATTGTCTCAAAG	TCAACATCCCGCACGTCTGTA	154
Cyclin E	AGCAGTCAGCCTTGGGATGAT	AGGCTCTGGGCGGTCTGATTT	139
Cyclin A	GGCCTTGTTCTCGAGACTTCTATTC	CCCAAATCTTCCAGCATGGTCTTA	182
Cdkn1a (P21)	GCAGACCAGCCTAACAGATTTCTA	TCCTGACCCACAGCAGAAGAAG	109
Cdkn1b (P27)	CAGACGTAAACAGCTCCGAATTAAG	GCGCAATGCTACATCCAATGCT	221
5-Htr1a	TCATCGGCTCCTTGGCGGTT	CCGGGGCGTCCTTTTGTTCA	233
5-Htr2a	ACCGACATGCCTCTCCATTCTTC	CAAAGGCCACCGGTACCCATACA	244
GR	AGGCCGGTCAGTGTTTTCT	CAATCGTTTCTTCCAGCACA	234
B2M	GTGCTTGCCATTCAGAAAACTCC	AGGTGGGTGGAACTGAGACA	136

Supplementary Table S3: Mann Whitney test results and corresponding p-values for comparisons between datasets which did not follow a normal distribution.

**Test and comparison**	**Mann Whitney U**	**p-value**
**FST**		
CT vs uCMS	5	0.0295
uCMS vs uCMS +FLX	5	0.0127
SCT		
CT vs uCMS	25	0.0067
uCMS vs uCMS +FLX	37	0.0072
**Cell Cycle exit**		
CT vs uCMS	20	0.3782
uCMS vs uCMS +FLX	3.5	0.4167
**Cyclin D1 DG expression**		
CT vs uCMS	1	0.0317
uCMS vs uCMS +FLX	7	0.1775
**P21 expression NSPs**		
CT vs uCMS	20	0.6620
uCMS vs uCMS +FLX	9	0.3290
**P57 expression NSPs**		
CT vs uCMS	5	0.3810
uCMS vs uCMS +FLX	2	0.400

Supplementary Table S4: Adapted table from FIMO motif search tool output for cyclin D1 promoter region (2000 kb upstream transcription start site (TSS)).

DATABASE AND MOTIFS

DATABASE sequences.fa
Database contains 1 sequences, 2000 residues

MOTIFS motifs.meme (DNA)

MOTIF	WIDTH	BEST POSSIBLE MATCH
1	15	AGAACAnnnTGTTCT

Random model letter frequencies (from non-redundant database):
A 0.275 C 0.225 G 0.225 T 0.275

SECTION I: HIGH-SCORING MOTIF OCCURENCES

- There were 1 motif occurences with a p-value less than 0.0001.
- The p-value of a motif occurrence is defined as the probability of a random sequence of the same length as the motif matching that position of the sequence with as good or better a score.
- The score for the match of a position in a sequence to a motif is computed by summing the appropriate entries from each column of the position-dependent scoring matrix that represents the motif.
- The q-value of a motif occurrence is defined as the false discovery rate if the occurrence is accepted as significant.
- The table is sorted by increasing p-value.

Motif	Sequence Name	Strand	Start	End	p-value	q-value	Matched Sequence
1	ENSRNOG00000020918 ENSRNOT00000088588	+	1611	1625	6.21e-05	0.247	AGTATAGGGTGTCCT
